# Supplementary material for: RE-AIM evaluation of a community-based vaccine education and communication program to improve human papillomavirus vaccine uptake in Tonga
Source: PLOS Glob Public Health. 2025 Nov 18;5(11):e0005467. doi: 10.1371/journal.pgph.0005467 (PMC12626289; doi:10.1371/journal.pgph.0005467)
Supplement: S1 Table — (DOCX) [file pgph.0005467.s001.docx]

**S1 Table: Effectiveness of Vaccine Champions training**

|  | **N*^*** | **Pre-training** | **Post-training** | **Diff (95% CI)** | **p** |
| --- | --- | --- | --- | --- | --- |
| **Knowledge about vaccines and communication** |  | **n (%)** | **n (%)** |  |  |
| Correctly identified false statement about vaccines | 14 | 7 (50.0) | 11 (78.6) | 28.6 (-2.2 to 59.4) | 0.125 |
| Correctly identified communication strategy | 19 | 11 (57.9) | 10 (52.6) | -5.3 (-41.4 to 30.9) | 1.000 |
| Correctly identified malaria vaccine not on EPI schedule | 18 | 13 (72.2) | 17 (94.4) | 22.2 (-2.5 to 47.0) | 0.125 |
| **Communication self-efficacy (very confident to…)** |  | **n (%)** | **n (%)** |  |  |
| Talk about side effects of vaccines | 20 | 13 (65.0) | 19 (95.0) | 30.0 (4.9 to 55.1) | 0.031 |
| Talk about the benefits of vaccines | 20 | 17 (85.0) | 19 (95.0) | 10.0 (-8.1 to 28.1) | 0.500 |
| Help someone find info about vaccines | 20 | 16 (80.0) | 18 (90.0) | 10.0 (-14.1 to 34.1) | 0.625 |
| Start a conversation with a hesitant person | 20 | 15 (75.0) | 19 (95.0) | 20.0 (-2.5 to 42.5) | 0.125 |
| **Vaccine confidence and trust (proportion who…)** |  | **n (%)** | **n (%)** |  |  |
| Trust the health system very much | 20 | 16 (80.0) | 18 (90.0) | 10.0 (-14.1 to 34.1) | 0.625 |
| Think HPV vaccine is very important for girls' health | 20 | 18 (90.0) | 19 (95.0) | 5.0 (-9.6 to 19.6) | 1.000 |
| Think routine vaccines are very important for child health | 20 | 19 (95.0) | 20 (100.0) | 5.0 (-9.6 to 19.6) | 1.000 |
| Think the HPV vaccine is very safe | 19 | 15 (78.9) | 18 (94.7) | 15.8 (-5.9 to 37.4) | 0.250 |
| Think routine vaccines are very safe | 18 | 15 (83.3) | 17 (94.4) | 11.1 (-9.0 to 31.2) | 0.500 |
| Think HPV vaccine works very well | 19 | 15 (78.9) | 18 (94.7) | 15.8 (-5.9 to 37.4) | 0.250 |
| Think routine vaccines work very well | 20 | 17 (85.0) | 19 (95.0) | 10.0 (-8.1 to 28.1) | 0.500 |

*^Total matched pre- and post-training responses for each question vary due to missing data*
