# Supplementary material for: RE-AIM evaluation of a community-based vaccine education and communication program to improve human papillomavirus vaccine uptake in Tonga
Source: PLOS Glob Public Health. 2025 Nov 18;5(11):e0005467. doi: 10.1371/journal.pgph.0005467 (PMC12626289; doi:10.1371/journal.pgph.0005467)
Supplement: S2 Table — (DOCX) [file pgph.0005467.s002.docx]

**S2 Table: Characteristics of community attendees completing post-session survey (N=269)**

| **Characteristic** | **Number (%)** |
| --- | --- |
| Island/group of origin |  |
| Tongatapu | 183 (68) |
| Vava’u | 44 (16) |
| Ha'apai | 22 (8) |
| 'Eua (Tongatapu group) | 17 (6) |
| The Niuas  Missing | 1 (1)  2 (1) |
| Gender |  |
| Female | 209 (78) |
| Male | 50 (19) |
| Prefer not to say/missing | 10 (3) |
