# Supplementary material for: RE-AIM evaluation of a community-based vaccine education and communication program to improve human papillomavirus vaccine uptake in Tonga
Source: PLOS Glob Public Health. 2025 Nov 18;5(11):e0005467. doi: 10.1371/journal.pgph.0005467 (PMC12626289; doi:10.1371/journal.pgph.0005467)
Supplement: Attachment — Submitted filename: Reviewer comments_8Jul25.docx [file pgph.0005467.s005.docx]

**Editor’s comments:**

1. Please include a complete copy of PLOS’ questionnaire on inclusivity in global research in your revised manuscript. Our policy for research in this area aims to improve transparency in the reporting of research performed outside of researchers’ own country or community. The policy applies to researchers who have travelled to a different country to conduct research, research with Indigenous populations or their lands, and research on cultural artefacts. The questionnaire can also be requested at the journal’s discretion for any other submissions, even if these conditions are not met. Please find more information on the policy and a link to download a blank copy of the questionnaire here: <https://journals.plos.org/globalpublichealth/s/best-practices-in-research-reporting>. Please upload a completed version of your questionnaire as Supporting Information when you resubmit your manuscript.

**Author response:** Thank you for drawing our attention to this questionnaire. We have completed it and submitted it with our resubmission.

2. In the ethics statement in the Methods, you have specified that verbal consent was obtained. Please provide additional details regarding how this consent was documented and witnessed, and state whether this was approved by the IRB.

**Author response:** Thank you for your query. We have added in additional details on the consent process with community attendees (Page 9-10). Our protocol stated that completing the community survey would be considered as providing consent as no identifying information was collected. For the community interviews, verbal consent was obtained. This process was approved by the ethics committees in Australia and Tonga.

“All participants provided written consent (for co-design participants and Vaccine Champions), verbal informed consent (for community attendee interviews), or implied consent (for community attendee surveys). Community surveys were anonymous, optional and did not collect any identifying data; surveys had a Participant Information Statement and completion of the survey was considered as consent to participate. In the case of verbal consent, the local research team provided community attendees with information outlining what the study was about, how their responses would be kept confidential, and what the data would be used for. All consent processes were finalised in collaboration with our local research partners and the Ministry of Health and approved by the ethics committee in Tonga and Australia.”

3. Please provide separate figure files in .tif or .eps format.

For more information about figure files please see our guidelines:
<https://journals.plos.org/globalpublichealth/s/figures>
<https://journals.plos.org/globalpublichealth/s/figures#loc-file-requirements>

**Author response:** Thank you for your comment. We have now included our figures as separate files in the requested formats.

4. In the online submission form, you indicated that [The de-identified datasets used and/or analysed during the current study are available from the corresponding author on reasonable request.].

All PLOS journals now require all data underlying the findings described in their manuscript to be freely available to other researchers, either
a. In a public repository,
b. Within the manuscript itself, or
c. Uploaded as supplementary information.

This policy applies to all data except where public deposition would breach compliance with the protocol approved by your research ethics board. If your data cannot be made publicly available for ethical or legal reasons (e.g., public availability would compromise patient privacy), please explain your reasons by return email and your exemption request will be escalated to the editor for approval. Your exemption request will be handled independently and will not hold up the peer review process, but will need to be resolved should your manuscript be accepted for publication. One of the Editorial team will then be in touch if there are any issues.

**Author response:** Thank you for your comment. We are in the process of uploading our de-identified quantitative data into a public repository through Figshare. When this is finalised, the data will be available at: [10.25374/MCRI.c.7902611](https://doi.org/10.25374/MCRI.c.7902611)

We have also requested an exemption for our qualitative data as making this publicly available would compromise the privacy of our study participants.

**Response to Reviewer 1**

**Comment:** Thank you for the opportunity to review this paper and learn about this program. HPV vaccination uptake is a critical public health issue, and this program is a feasibly and potentially highly effective intervention. I have a few comments and questions, primarily regarding the quantitative methods:

**Author response:** Thank you for your kind review of this paper. We appreciate your comments and have addressed them individually below.

**Abstract**
**Comment:** The word diverse is used twice in the sentence starting “Between March and December 2023” – consider more varied language

**Author response:** Thank you for picking this up, we have removed one of the instances of “diverse”.

**Comment:** Can you clarify when the program was actually run (dates), what data were collected, and who the participants were? The current abstract makes it sound as though the main focus was the co-design workshop.

**Author response:** Thank you for this suggestion. We have amended this section of the abstract to read as follows:

“In March 2023 we conducted a co-design workshop with government and non-government stakeholders to establish program features. Diverse Vaccine Champions were trained in June to run vaccine information sessions in their communities until December 2023. We used the RE-AIM framework to evaluate the program through surveys and interviews with co-design participants, Vaccine Champions, and community members.”

**Introduction**
**Comment:** In the intro, there’s a focus on the intention-uptake gap, but this intervention seems to focus on intention. I recognize that you couldn’t measure uptake, but can you comment a bit on why intention and programs like the Vaccine Champion program, are still relevant?

**Author response:** Thank you for your question. Community engagement programs including those that train vaccine champions have been shown to increase both vaccine intention and vaccine uptake. The Vaccine Champions program targets multiple drivers of vaccine uptake; while were not able to measure vaccine uptake, multi-level interventions like this program have been shown to be the most effective at improving HPV vaccine uptake. We have added some text to the introduction on page 5 to make this more explicit:

“Systematic reviews also support the use of multi-level interventions to increase HPV vaccine uptake (17).

This study aimed to evaluate the Vaccine Champions program, a comprehensive community-based vaccine education and communication skills training program for diverse community advocates to improve acceptance and uptake of the HPV vaccine in Tonga. The program targets multiple drivers of vaccine uptake by improving knowledge, addressing community concerns and misinformation, capitalising on social norms, and reminding people about vaccination (13, 17).”

We have also rewritten the sentence on the intention-uptake gap in Tonga to make it clearer that while caregivers in Tonga had high vaccine intention, most had not heard of the HPV vaccine before the vaccine introduction (page 4).

“Prior to vaccine introduction, a survey of caregivers in Tonga indicated that only 37% had heard of the HPV vaccine (10). After learning about it through the survey, 92% stated they intended to vaccinate their child against HPV (10), but by February 2023 the Ministry of Health estimated that only 20% of eligible girls had received the vaccine (12), leading them to seek strategies to bridge the intention-uptake gap.

**Methods**
**Comment:** Is there a citation for roll out in Vietnam? (p5 line 92)

**Author response:** Our findings from the program evaluation in Vietnam have been submitted for publication but have not yet been published; we are therefore unable to add in a citation.

**Comment:** Was any specific type of voting using in co-design workshop (ranked choice, consensus-building methods)?

**Author response:** Thank you for your question. We have added in some details of the type of voting used in the co-design workshop on page 6 of the manuscript:

“Participants then voted on program features using dot voting to prioritise from a range of options.”

**Comment:** The data collection section is slightly confusing. It goes from a high level overview of the phases, to data collection among the vaccine champions, then finishes with the co-design participants. Can you reorganize so it follows the structure of the table and the progress of the study phase? I’d suggest [study phase], [participants], [data collection methods].

**Author response:** Thank you for this suggestion. We have reorganised this section according to your recommendation:

***“Phase 1: Co-design participants***

Co-design participants were invited to complete a survey in English immediately after participating in the co-design workshop. Interviews with co-design participants involved in program implementation were conducted in English by the first author.

***Phase 2: Vaccine Champions***

Vaccine Champions completed surveys before and immediately after training, measuring knowledge, vaccine confidence and trust, and communication self-efficacy, as in our Fiji program evaluation (13). Knowledge questions were developed by the research team. Vaccine confidence and trust were measured through seven items adapted from the WHO tools for understanding the behavioural and social drivers of vaccination (19). We measured communication self-efficacy with four questions adapted from Bandura’s self-efficacy scale (22).

Vaccine Champions completed a reflection form after each community session, capturing their experiences and personal reflections, and recording the number and type of community members invited to and attending the session. Champions measured attendees’ intention to get the HPV vaccine for their daughters with a show of hands before and after each session. All data collection forms for Champions were available in Tongan and interviews were conducted by the local research team in Tongan or English.

***Phase 3: Community attendees***

Community attendees completed a short paper survey in Tongan post-session, collecting demographic details and satisfaction. Self-reported changes in vaccine confidence and trust were measured with four questions adapted from the WHO BeSD tools (19), using a 3-point scale from “less than before” to “more than before”. Community attendees were also asked if they would like to take part in an interview. If they did, they had the option of adding their contact details to the Champions’ reflection form and were later contacted and invited to take part in a qualitative interview in Tongan, conducted by Tongan members of the research team.”

**Results**
**Comment:** What were the results of the voting on program characteristics in the co-design-workshop?

**Author response:** We appreciate your thoughtful question and have added in the following text on page 10 of the manuscript:

“The results of the voting by co-design participants were a program focused on the HPV vaccine; a two-day training workshop for Vaccine Champions; a more diverse range of Champions with smaller numbers in each group; and most Champions to come from the main island of Tongatapu.”

**Comment:** What are disability groups, in this context?

**Author response:** Thank you for your question. In this context, disability groups refer to people who live with disability and work to empower others living with disability. We have updated the text on page 12 to make this clearer:

“Champions included teachers; school nurses; local government officials; staff from non-government organisations; representatives from disabled people’s organisations; and members of youth, sports, and faith groups.”

**Comment:** What was the average size of the vaccine info sessions? Any variability by region/vaccine champion/other factors?

**Author response:** Thank you for your question. We have added in a sentence on page 15 stating the mean number and range of attendees. We do provide information about the gender and location of community attendees who completed a survey in the supplemental materials (S2 Table), however we do not have data from all attendees and given the small sample size we did not disaggregate by region or Vaccine Champion session.

“Sessions had a mean of 22 attendees, with a range of less than five people to 55 community members present.”

**Comment:** Did you consider conducting any statistical analyses of your outcomes by sociodemographic characteristics?

**Author response:** Thank you for your question. While we did have some sociodemographic data on the Vaccine Champions and community attendees, we did not disaggregate the data by these characteristics due to the small sample sizes – only 20 Champions completed both pre- and post-surveys.

**Comment:** Did you consider the effect of clustering by vaccine champion (e.g., participants in sessions run by the same champion may be more similar to each other than participants in sessions run by a different vaccine champion)?

**Author response:** Thank you for this insightful question, we agree that participants in sessions run by the same Champion may be more similar to those in sessions run by different Champions. We did not consider the effect of clustering by Vaccine Champion given the small sample sizes and the low response rate for the community surveys. The community surveys were completed by those attending a range of Champions’ sessions (20/23 Champions). We have added in a sentence to clarify this on page 16:

“Community members who completed surveys attended sessions run by 20 different Champions, with only three Champions who ran sessions having no surveys completed about at least one of their sessions.”

We have also included more information about response bias in the limitations section of the manuscript.

**Discussion**
**Comment:** In the on-going program, will there be any additional compensation for vaccine champions as suggested by participants?

**Author response:** Thank you for your query. Yes, in the on-going program Vaccine Champions will be provided with monetary incentives for running community information sessions. We have added this information at the bottom of page 9:

“The continuation of the Vaccine Champions program in Tonga in 2025 will include monetary incentives for Champions.”

**Comment:** Thank you for the discussion of gender-inclusive HPV vaccine policies. In Tonga, who typically holds decision-making power for medical decisions for children (mothers vs fathers)? Could male vaccine champions be useful in speaking with fathers?

**Author response:** Thank you for raising this important point. We have added in a sentence clarifying that while mothers are the main decision-makers around childhood vaccines in Tonga, fathers also have an important role to play (page 19):

“While mothers tend to the primary decision-maker about childhood immunisations in Tonga, fathers do still have an important role to play in vaccine decision-making and sharing information with other fathers (10, 32).”

**Comment:** A key limitation is social desirability bias, both in how people respond to survey questions and who even responds in the first place. Can you discuss the impact of low participation in the post-event surveys on your results?

**Author response:** Thank you for your comment. We agree that social desirability and response bias are challenging with any data collection and we have added in a sentence on page 21 to clarify that this may have positively skewed our evaluation results:

“Our translated surveys were reviewed by the local research team to maximise understanding but self-reported measures of confidence and satisfaction may be impacted by social desirability bias. The combination of response bias and social desirability bias may mean that those who completed the surveys had a more positive view of the program than those who did not. This is particularly true for the community surveys, where only 23% of attendees completed a survey. All Champions were asked to distribute surveys at their sessions and survey participants attended sessions run by most Champions. However, it is possible that those whose sessions went well were more likely to hand out the survey to community attendees.”
