# Supplementary material for: RE-AIM evaluation of a community-based vaccine education and communication program to improve human papillomavirus vaccine uptake in Tonga
Source: PLOS Glob Public Health. 2025 Nov 18;5(11):e0005467. doi: 10.1371/journal.pgph.0005467 (PMC12626289; doi:10.1371/journal.pgph.0005467)
Supplement: Attachment — Submitted filename: Response to reviewers_Oct25.docx [file pgph.0005467.s006.docx]

**Reviewer 1**

**Comment:** Thank you for the thorough and thoughtful response to my comments. I believe this paper makes an important contribution to the literature on HPV vaccination uptake, and I appreciate that the level of detail you have provided makes it possible for others in different contexts to draw on your experiences when building their own programs.

**Author response:** Thank you for your kind feedback on our manuscript.

**Reviewer 2**

**Comment:** The purpose of this study was to evaluate an HPV vaccine uptake campaign in Tonga. The manuscript could be strengthened by clarifying differences in evaluating program planning/adaptation, facilitator training, and implementation of the program itself.

**Author response:** Thank you for your comment. This paper outlines the findings from a mixed methods evaluation of the Vaccine Champions program. The program includes all three phases mentioned: the co-design (planning/adaptation), the training of Champions (facilitator training) and the community sessions (downstream implementation). We therefore evaluated all three of these phases. We have tried to clarify the distinction between the description of the program phases and their evaluation with the following changes:

Page 5 (lines 96-99)

“This paper presents the results of a mixed methods evaluation of the Vaccine Champions program, focusing on how each phase of the program was implemented and its outcomes. The next section provides an overview of the three phases of the program itself, then moves on to details of the evaluation methods.”

Page 7 (lines 155-161)

**“Evaluation design**

We evaluated program implementation and impact using the RE-AIM framework, assessing intervention reach, effectiveness, adoption, implementation, and maintenance (1, 2). This framework is commonly used to evaluate public health and behaviour change interventions (23). It enables understanding of individual and community-level outcomes, as well as exploring how and why the intervention works (20). The RE-AIM domains measured for each program phase are outlined in Table 1.”

**Comment:** The introduction focuses exclusively on cervical cancer, although HPV causes multiple types of cancers. The first paragraph also only mentions girls being vaccinated, reinforcing the feminization of HPV. Why is the HPV vaccine not available for boys in Tonga?

**Author response:** Thank you for your comment. We have added in a line to the introduction (line 56) highlighting the benefits of the HPV vaccine in protecting against other forms of cancer:

“All currently available HPV vaccines are highly effective against the two virus types causing over 70% of cervical cancer cases, and additionally protect against many other forms of cancer (3).”

As in many low- and middle-income countries, the HPV vaccine is available only for girls in Tonga at this stage. This is due to the cost of the vaccine and the limited resources available for the vaccination program. The World Health Organization recommends the HPV vaccine for adolescent girls due to the significant burden of cervical cancer in low- and middle-income countries. I do agree that it is important to move towards gender neutral HPV vaccination (as highlighted in the discussion section lines 422-430), however this is not yet the reality in many settings.

**Comment:** When the Vaccine Champions program was evaluated in Fiji and Vietnam – was this also in relation to COVID-19 or HPV vaccine? Or, was this the first time it had been used for HPV vaccine?

**Author response:** Thank you for highlighting this. We have added in some text to clarify that the program focused on COVID-19 and routine childhood vaccines in Fiji and Viet Nam, and that it was the first time it had focused on the HPV vaccine in Tonga:

“It was initially developed in Australia to facilitate COVID-19 vaccine uptake (4), and has since been implemented and evaluated in Fiji (2) and Viet Nam with a focus on COVID-19 and routine childhood vaccines. In 2023 in partnership with the Tonga Ministry of Health, UNICEF Pacific, and the Tupou Tertiary Institute, we adapted, implemented, and evaluated the program in Tonga. This was the first time the program had focused on the HPV vaccine.”

**Comment:** There could be more of a focus in the title/abstract/introduction that this program was also adapted, not just evaluated.

**Author response:** Thank you for your suggestion. This manuscript describes the program evaluation (implementation and outcomes – we have clarified this as per your suggestion in a later comment), but does not specifically focus on program adaptation. As noted above, the Vaccine Champions program has 3 key phases: co-design (where the program is adapted to the setting), training for Vaccine Champions, and phase 3 where Champions run sessions in their communities. Adaptation of the program is therefore an inherent part of the program itself. We have updated the abstract of our paper to make this clearer:

“In March 2023 we conducted a co-design workshop with government and non-government stakeholders to adapt the program and establish key features. Diverse Vaccine Champions were trained in June to run vaccine information sessions in their communities until December 2023. We used the RE-AIM framework to evaluate program implementation and impact through surveys and interviews with co-design participants, Vaccine Champions, and community members.”

**Comment:** The introduction states that the purpose of this study was to evaluate the Vaccine Champions Program – but the methods and results also include a lot of program planning.

**Author response:** Thank you for bringing this to our attention. We have updated the final paragraph of the introduction to make it clear that this paper presents findings from an evaluation of the implementation and impact of the Vaccine Champions program in Tonga. While the paper also describes the program itself, the primary focus is on the results of the evaluation.

“This study aimed to evaluate the implementation and impact of the Vaccine Champions program, a comprehensive community-based vaccine education and communication skills training program for diverse community advocates to improve acceptance and uptake of the HPV vaccine in Tonga. The program targets multiple drivers of vaccine uptake by improving knowledge, addressing community concerns and misinformation, capitalising on social norms, and reminding people about vaccination (2, 4). We used the RE-AIM framework to measure and describe reach, effectiveness, adoption, implementation, and maintenance.”

**Comment:** It is mentioned that an implementation science approach is used- more information needed about that.

**Author response:** Thank you for your comment. The implementation science approach we used is the RE-AIM framework. To make it clear that this framework was used to evaluate the program, we have changed the wording of this sentence:

“We used the RE-AIM framework to measure and describe reach, effectiveness, adoption, implementation, and maintenance.”

**Comment:** More information about what co-design is would be helpful.

**Author response:** Thank you for this suggestion. We have added additional information about the co-design workshop and what it involved:

“The core components of the program were predetermined, however the features decided at the co-design workshop included vaccine focus, who should be a Vaccine Champion, how to incentivise Champions and length of Champions’ training.”

**Comment:** It seems that phase 1 and 2 are more about program planning than program evaluation

**Author response:** Please see comments above regarding describing how each phase was implemented and its outcomes.

**Comment:** Phase 2 mentions training, but not what the training entailed. Was this the Vaccine Champions themselves being trained,?

**Author response:** Thank you for your question. Yes, the training workshop was for the Vaccine Champions. As outlined on page 6 (lines 118-128), the training included information about vaccines and vaccine-specific communication skills. We have updated the text to reflect this more clearly:

“Phase 2 was a two-day training workshop in Tongatapu to train Vaccine Champions, delivered in English with local staff from Tupou Tertiary Institute and the Ministry of Health translating as required. Training materials were available in Tongan and English and were provided online for a participant with a visual impairment to process with appropriate software. Training topics included the HPV vaccine, routine childhood vaccines, vaccine communication skills including conversations with vaccine hesitant people, addressing misinformation, and practical tips on hosting community information sessions. Champions practiced communication skills through structured role plays.”

**Comment:** Phase 3 mentions community attendees – are they attending the Vaccine Champions program or the co-design workshop?

**Author response:** Thank you for the question. We have added in a sentence on page 7 to clarify that the community attendees were people attending Champions’ sessions:

“The community members who attended a Vaccine Champion’s information session were included as “community attendees” in the program evaluation.”

**Comment:** A clearer differentiation between adapting the program using co-design, training the vaccine champions, and then evaluating the implementation of the program itself would strengthen the paper.

**Author response:** Thank you for your comment. The co-design, training and community sessions are all phases of the program itself. We evaluated all three phases of the program – this paper describes the evaluation findings rather than focusing on the adaptation process (which is inherent in the program itself). We have added the following text at the start of the Methods section to make this clearer:

“This paper presents the results of a mixed methods evaluation of the Vaccine Champions program, focusing on how each phase of the program was implemented and its outcomes. The next section provides an overview of the three phases of the program itself, then moves on to details of the evaluation methods.”

**Comment:** In Table 1 – it seems that you are evaluating both the development of the program, the training of the Vaccine champions, and the implementation of the program – is that correct? (For example, with co-design participants it says the RE-AIM domain of implementation was included, but it’s unclear what program was implemented during this co-design)

**Author response:** Thank you for your question. The Vaccine Champions includes the co-design process (where the program is adapted to the local context) as one of its core components. We therefore evaluate all three phases of the program: the co-design, the Champions training, and the Champions’ sessions. The text added to respond to the previous comment hopefully helps to clarify this and we have added a column to Table 1 showing which program phase corresponds with which RE-AIM domains.

**Comment:** It is not clear how the vaccine champions were trained – were they given the same program that they would then be teaching to others?

**Author response:** Thank you for the question. Vaccine Champions could use the knowledge and skills they gained from the training to share information with their communities however worked for them. We have added a sentence on page 6 (lines 125-128) to clarify this:

“They were given some suggestions for what to cover in a community information session and were encouraged to use their vaccine knowledge and communication skills to share information about the HPV vaccine with their community in any way that worked for them.”

Lines 135-138 also highlight the different ways that Champions could choose to run their sessions. These sessions were meant to be tailored to the individual Champion’s preferences.

**Comment:** In table one it mentions “Implementation: adaptations made to program implementation” – what does this mean?

**Author response:** Thank you for the question. We agree that this is unclear and have updated the phrase to match the co-design participants in Table 1:

“Implementation: overall program implemented as intended”

Here we looked at implementation fidelity, so the extent to which the program was implemented as it was intended to be. From the perspectives of the Vaccine Champions, that would be things like whether their sessions went as they planned them to go and any barriers they faced to running sessions.

**Comment:** I am a little bit confused about RE-AIM being applied to the adaptation of the program and the training of the champions as well as to the program itself. I know that RE-AIM has been used as a process framework to plan implementation and adaptations – so is it being used here for both process and outcome evaluation? Clarifying that in the study design would be helpful. Currently it is worded in a way that suggests outcome evaluation only.

**Author response:** We appreciate your suggestion and have modified the final paragraph of the introduction accordingly:

“This study aimed to evaluate the implementation and impact of the Vaccine Champions program, a comprehensive community-based vaccine education and communication skills training program for diverse community advocates to improve acceptance and uptake of the HPV vaccine in Tonga. The program targets multiple drivers of vaccine uptake by improving knowledge, addressing community concerns and misinformation, capitalising on social norms, and reminding people about vaccination (2, 4). We used the RE-AIM framework to measure and describe reach, effectiveness, adoption, implementation, and maintenance.”

**Comment:** “We evaluated the three program phases using qualitative and quantitative methods (Table 1).” – would be helpful to break this down into what is program planning, implementation, process and outcome eval.

**Author response:** Thank you for your suggestion. In response to the previous comment, we have clarified that the evaluation was of program implementation and impact. Given our use of the RE-AIM framework to guide the evaluation, we believe that splitting the outcomes by the RE-AIM domains fits better with our paper than categorising them by whether they relate to the process or outcome evaluation.

**Comment:** How many team members coded each interview? How were themes determined within each area of RE-AIM?

**Author response:** Thank you for the question. We have added in the requested details:

“Qualitative data (transcripts, reflection forms and field notes) were transcribed and translated where necessary, then analysed thematically by the first author using NVivo version 12 (5). The research team discussed and agreed upon the coding framework for analysis, grouped the codes into themes and then mapped the codes to the different dimensions of the RE-AIM framework.”

**Comment:** In the results, it is mentioned that “one participant felt…”. Does this represent a theme or did only one participant feel that way? Unclear how it was decided what quotes and views are included.

**Author response:** Thank you for your question. We have added some text in the data analysis section to clarify that the qualitative data was collected to supplement the quantitative data and provide more depth. We also include more details on the analysis and how quotes were chosen. The data in the results has also been amended to clearly show whether the quotes reflect common or divergent perspectives.

**Data analysis**

“Qualitative data were collected to supplement the quantitative findings and provide additional in-depth insights (26). Qualitative data (transcripts, reflection forms and field notes) were transcribed and translated where necessary, then analysed by the first author with NVivo version 12, using a deductive content approach (27). The research team discussed and agreed upon the coding framework for analysis, then mapped the codes to the different dimensions of the RE-AIM framework. Here we present quotes that are illustrative of common perspectives from across the qualitative findings as well as individual divergent perspectives.”

**Results**

While most co-design participants were satisfied with the length, two survey participants felt the session could have been longer, to include more small group discussions. In an end of program interview, one co-design participant from the Ministry of Health felt it would have been beneficial to have the most senior staff from the Ministry attend the entire workshop to maximise high-level program support. Another interviewee thought there was good representation from health and non-health stakeholders.”

Vaccine Champions reported their confidence to communicate about vaccines increased after the training in all four areas assessed (Figure 2c). This was also reflected in the interviews, with Champions describing how the training gave them confidence to speak about sensitive issues in front of a group.

Champions also spoke highly about the training in interviews, highlighting that it was informative, included the right people and addressed key community concerns. One Champion felt it would have been better to only include women in the training given the sensitive nature of the HPV vaccine and another thought the training could have been longer.

We interviewed several Champions who did not run multiple sessions. The barriers they highlighted to running one or more sessions included lack of time, low interest from parents, and not being able to obtain approval from school principals to run sessions with students.”

**Comment:** “Over the study period, HPV vaccine coverage increased by 12 % and the 480 Ministry of Health believed the program had been well received in the community.” – need a citation for this

**Author response:** Thank you for highlighting this. We have added in a citation for the HPV vaccine coverage in Tonga. These data are from the Tonga Ministry of Health and are based on their monitoring of national vaccine coverage.
